# Supplementary material for: Beta-Blocker and Renin–Angiotensin System Inhibitor Combination Therapy in Patients with Acute Myocardial Infarction and Prediabetes or Diabetes Who Underwent Successful Implantation of Newer-Generation Drug-Eluting Stents: A Retrospective Observational Registry Study
Source: J Clin Med. 2020 Oct 27;9(11):3447. doi: 10.3390/jcm9113447 (PMC7692957; doi:10.3390/jcm9113447)
Supplement: Supplementary file 1 [file jcm-09-03447-s001.zip › jcm-958201-supplementary.docx]

**Table S1.** Baseline clinical, laboratory, angiographic, and procedural characteristics of the total study population.

| **Variables** | **Normoglycemia**  **(*n* = 2460)** | |  | **Prediabetes**  **(*n* = 2907)** | | | **Diabetes**  **(*n* = 4099)** | | |
| --- | --- | --- | --- | --- | --- | --- | --- | --- | --- |
|  | **BB+RASI (+)**  **A1**  **(*n* = 2217)** | **BB+RASI (-)**  **A2**  **(*n* = 243)** | ***p* value** | **BB+RASI (+)**  **B1**  **(*n* = 2601)** | **BB+RASI (-)**  **B2**  **(*n* = 306)** | ***p* value** | **BB+RASI (+)**  **C1**  **(*n* = 3682)** | **BB+RASI (-)**  **C2**  **(*n* = 417)** | ***p* value** |
| Men, *n* (%) | 1803 (81.3) | 190 (78.2) | 0.237 | 1995 (76.7) | 209 (68.3) | 0.001 | 2588 (70.3) | 267 (64.0) | 0.008 |
| Age (years) | 60.4 ± 13.0 | 64.3 ± 13.4 | <0.001 | 62.6 ± 12.4 | 66.7 ± 13.4 | <0.001 | 63.7 ± 11.5 | 67.2 ± 11.8 | <0.001 |
| LVEF (%) | 53.2 ± 10.3 | 49.9 ± 13.1 | <0.001 | 53.2 ± 10.4 | 49.7 ± 13.6 | <0.001 | 51.7 ± 11.3 | 47.3 ± 13.7 | <0.001 |
| ≤40%, *n* (%) | 182 (8.2) | 43 (17.7) | <0.001 | 231 (8.9) | 61 (19.9) | <0.001 | 492 (13.4) | 117 (28.1) | <0.001 |
| BMI (kg/m^2^) | 23.9 ± 3.1 | 23.4 ± 3.1 | 0.030 | 24.4 ± 3.2 | 23.2 ± 3.4 | <0.001 | 24.5 ± 3.2 | 23.9 ± 3.6 | 0.004 |
| SBP (mmHg) | 134.1 ± 27.7 | 119.8 ± 25.1 | <0.001 | 131.6 ± 27.6 | 122.8 ± 28.1 | <0.001 | 132.8 ± 27.9 | 123.8 ± 29.5 | <0.001 |
| DBP (mmHg) | 82.3 ± 16.6 | 74.7 ± 14.3 | <0.001 | 80.2 ± 16.5 | 75.4 ± 15.8 | <0.001 | 79.7 ± 16.2 | 75.4 ± 16.8 | <0.001 |
| STEMI, *n* (%) | 1346 (60.7) | 150 (61.7) | 0.758 | 1578 (60.7) | 185 (60.5) | 0.943 | 1952 (53.0) | 232 (55.6) | 0.309 |
| Primary PCI, *n* (%) | 1303 (96.8) | 143 (95.3) | 0.341 | 1530 (97.0) | 174 (94.1) | 0.038 | 1884 (96.5) | 218 (94.0) | 0.053 |
| NSTEMI, *n* (%) | 871 (39.3) | 93 (38.3) | 0.758 | 1023 (39.3) | 121 (39.5) | 0.943 | 1730 (47.0) | 185 (44.4) | 0.309 |
| PCI within 24 hours | 774 (88.9) | 78 (84.3) | 0.153 | 887 (86.7) | 101 (83.2) | 0.327 | 1480 (85.5) | 153 (82.7) | 0.299 |
| Hypertension, *n* (%) | 930 (41.9) | 83 (34.2) | 0.019 | 1163 (44.7) | 130 (42.5) | 0.458 | 2210 (60.0) | 259 (62.1) | 0.409 |
| Dyslipidemia, *n* (%) | 192 (8.7) | 12 (4.9) | 0.046 | 294 (11.3) | 32 (10.5) | 0.657 | 529 (14.4) | 52 (12.5) | 0.292 |
| Previous MI, *n* (%) | 64 (2.9) | 8 (3.3) | 0.722 | 55 (2.1) | 12 (3.9) | 0.046 | 166 (4.5) | 13 (3.1) | 0.188 |
| Previous PCI, *n* (%) | 84 (3.8) | 10 (4.1) | 0.801 | 117 (4.5) | 16 (5.2) | 0.562 | 277 (7.5) | 27 (6.5) | 0.439 |
| Previous CABG, *n* (%) | 5 (0.2) | 1 (0.4) | 0.577 | 3 (0.1) | 0 (0.0) | 0.552 | 22 (0.6) | 4 (1.0) | 0.378 |
| Previous CVA, *n* (%) | 97 (4.4) | 17 (7.0) | 0.065 | 123 (4.7) | 16 (5.2) | 0.671 | 275 (7.5) | 45 (10.8) | 0.017 |
| Previous HF, *n* (%) | 9 (0.4) | 1 (0.4) | 0.990 | 17 (0.7) | 8 (2.6) | <0.001 | 44 (1.2) | 12 (2.9) | 0.005 |
| Current smokers, *n* (%) | 1028 (46.4) | 118 (48.6) | 0.516 | 1228 (47.2) | 124 (40.5) | 0.026 | 1464 (39.8) | 132 (31.7) | 0.001 |
| Peak CK-MB (mg/dL) | 134.4 ± 208.2 | 187.3 ± 278.8 | <0.001 | 142.0 ± 205.6 | 163.6 ± 198.4 | 0.074 | 101.2 ± 137.9 | 128.6 ± 158.8 | <0.001 |
| Peak troponin-I (ng/mL) | 46.6 ± 81.5 | 59.8 ± 119.8 | 0.137 | 49.8 ± 138.4 | 50.6 ± 89.1 | 0.897 | 45.6 ± 144.8 | 61.2 ± 196.8 | 0.069 |
| Blood glucose (mg/dL) | 136.6 ± 49.9 | 152.4 ± 67.4 | 0.001 | 146.5 ± 45.7 | 157.2 ± 58.0 | 0.002 | 222.8 ± 96.3 | 245.3 ± 119.4 | <0.001 |
| Hemoglobin A1c (%) | 5.33 ± 0.43 | 5.30 ± 0.62 | 0.403 | 5.96 ± 0.21 | 5.98 ± 0.21 | 0.143 | 7.81 ± 2.87 | 7.88 ± 3.40 | 0.673 |
| NT-ProBNP (pg/mL) | 178.0 (52.0-801.5) | 299.0 (58.0-2645.0) | <0.001 | 223.0 (56.0-919.0) | 467.0 (101.5-2229.0) | 0.001 | 348.5 (78.8-1750.5) | 1124.0 (163.5-6746.5) | <0.001 |
| hs-CRP (mg/dL) | 5.7 ± 31.2 | 13.2 ± 56.5 | 0.007 | 8.9 ± 55.9 | 15.4 ± 49.3 | 0.060 | 9.1 ± 43.6 | 22.4 ± 66.0 | <0.001 |
| Serum creatinine (mg/L) | 0.99 ± 0.87 | 1.04 ± 0.44 | 0.192 | 1.02 ± 1.02 | 1.35 ± 3.95 | 0.001 | 1.13 ± 1.08 | 1.45 ± 1.58 | <0.001 |
| eGFR (mL/min/1.73 m^2^) | 92.1 ± 33.9 | 84.1 ± 31.8 | <0.001 | 90.4 ± 37.3 | 84.4 ± 37.8 | 0.008 | 86.6 ± 44.6 | 74.8 ± 37.5 | <0.001 |
| <60 mL/min/1.73 m^2^ | 242 (10.9) | 53 (21.8) | <0.001 | 349 (13.4) | 66 (21.6) | 0.001 | 779 (21.2) | 148 (35.5) | <0.001 |
| Total cholesterol (mg/dL) | 183.0 ± 40.1 | 172.1 ± 42.5 | <0.001 | 190.6 ± 42.9 | 184.6 ± 46.2 | 0.033 | 180.6 ± 48.9 | 172.5 ± 48.4 | 0.002 |
| Triglyceride (mg/L) | 120.8 ± 91.8 | 102.8 ± 67.4 | <0.001 | 138.3 ± 111.8 | 111.1 ± 85.1 | <0.001 | 154.5 ± 138.6 | 141.0 ± 117.7 | 0.034 |
| HDL cholesterol (mg/L) | 44.5 ± 144 | 45.5 ± 25.7 | <0.001 | 43.9 ± 15.8 | 44.2 ± 20.3 | 0.808 | 42.0 ± 14.2 | 41.0 ± 14.8 | 0.175 |
| LDL cholesterol (mg/L) | 116.4 ± 36.1 | 109.9 ± 41.6 | 0.022 | 122.6 ± 49.3 | 120.2 ± 39.9 | 0.358 | 111.4 ± 38.8 | 104.6 ± 41.7 | 0.002 |
| Diabetes management |  |  |  |  |  |  |  |  |  |
| Diet |  |  |  |  |  |  | 314 (8.5) | 28 (6.7) | 0.204 |
| Oral agent |  |  |  |  |  |  | 2305 (62.6) | 266 (63.8) | 0.635 |
| Insulin |  |  |  |  |  |  | 173 (4.7) | 27 (6.5) | 0.111 |
| untreated |  |  |  |  |  |  | 890 (24.2) | 96 (23.0) | 0.872 |
| Discharge medications |  |  |  |  |  |  |  |  |  |
| Aspirin, *n* (%) | 2205 (99.5) | 218 (89.7) | <0.001 | 2587 (99.5) | 266 (86.9) | <0.001 | 3669 (99.6) | 367 (88.0) | <0.001 |
| Clopidogrel, *n* (%) | 1774 (80.0) | 200 (82.3) | 0.395 | 2229 (85.7) | 264 (86.3) | 0.785 | 3166 (86.0) | 344 (82.5) | 0.054 |
| Ticagrelor, *n* (%) | 281 (12.7) | 20 (8.2) | 0.045 | 235 (9.0) | 22 (7.2) | 0.282 | 282 (7.7) | 34 (8.2) | 0.720 |
| Prasugrel, *n* (%) | 147 (6.6) | 8 (3.3) | 0.042 | 122 (4.7) | 12 (3.9) | 0.665 | 194 (5.3) | 27 (6.5) | 0.301 |
| Cilostazole, *n* (%) | 304 (13.7) | 23 (9.5) | 0.073 | 495 (19.0) | 38 (12.4) | 0.005 | 749 (20.3) | 44 (10.6) | <0.001 |
| CCBs, *n* (%) | 86 (3.9) | 21 (8.6) | 0.001 | 96 (3.7) | 25 (8.2) | <0.001 | 219 (5.9) | 29 (7.0) | 0.414 |
| Lipid lowering agents | 2267 (93.7) | 190 (78.2) | <0.001 | 2431 (93.5) | 247 (80.7) | <0.001 | 3320 (90.2) | 340 (81.5) | <0.001 |
| IRA |  |  |  |  |  |  |  |  |  |
| Left main, *n* (%) | 37 (1.7) | 7 (2.9) | 0.176 | 31 (1.2) | 8 (2.6) | 0.041 | 65 (1.8) | 15 (3.6) | 0.010 |
| LAD, *n* (%) | 1152 (52.0) | 118 (48.6) | 0.314 | 1337 (51.4) | 150 (49.0) | 0.430 | 1707 (46.4) | 207 (49.6) | 0.203 |
| LCx, *n* (%) | 371 (16.7) | 39 (16.0) | 0.786 | 439 (16.9) | 42 (13.7) | 0.160 | 638 (17.3) | 62 (14.9) | 0.217 |
| RCA, *n* (%) | 657 (29.6) | 79 (32.5) | 0.353 | 794 (30.5) | 106 (34.6) | 0.141 | 1272 (34.5) | 133 (31.9) | 0.280 |
| Treated vessel |  |  |  |  |  |  |  |  |  |
| Left main, *n* (%) | 55 (2.5) | 11 (4.5) | 0.061 | 62 (2.4) | 13 (4.2) | 0.052 | 108 (2.9) | 21 (5.0) | 0.026 |
| LAD, *n* (%) | 1342 (60.5) | 138 (56.8) | 0.258 | 1577 (60.6) | 168 (54.9) | 0.053 | 2155 (58.5) | 246 (59.0) | 0.856 |
| LCx, *n* (%) | 563 (25.4) | 51 (21.0) | 0.132 | 666 (25.6) | 77 (25.2) | 0.867 | 1039 (28.2) | 114 (27.3) | 0.705 |
| RCA, *n* (%) | 783 (35.3) | 91 (37.4) | 0.510 | 961 (36.9) | 118 (38.6) | 0.580 | 1560 (42.4) | 161 (38.6) | 0.140 |
| ACC/AHA lesion type |  |  |  |  |  |  |  |  |  |
| Type B1, *n* (%) | 294 (13.3) | 25 (10.3) | 0.190 | 352 (13.5) | 32 (10.5) | 0.133 | 458 (12.4) | 57 (13.7) | 0.473 |
| Type B2, *n* (%) | 767 (34.6) | 59 (24.3) | 0.001 | 853 (32.8) | 94 (30.7) | 0.464 | 1284 (34.9) | 103 (24.7) | <0.001 |
| Type C, *n* (%) | 983 (44.3) | 118 (48.6) | 0.209 | 1145 (44.0) | 121 (69.5) | 0.135 | 1635 (44.4) | 191 (45.8) | 0.586 |
| Extent of CAD |  |  |  |  |  |  |  |  |  |
| 1-vessel, *n* (%) | 1219 (55.0) | 139 (57.2) | 0.659 | 1396 (53.7) | 151 (49.3) | 0.101 | 1605 (43.6) | 170 (40.8) | 0.216 |
| 2-vessel, *n* (%) | 655 (29.5) | 69 (28.4) | 0.709 | 785 (30.2) | 93 (30.4) | 0.939 | 1215 (33.0) | 140 (33.6) | 0.813 |
| ≥ 3-vessel, *n* (%) | 343 (15.5) | 35 (14.4) | 0.661 | 420 (16.1) | 62 (20.3) | 0.067 | 862 (23.4) | 107 (25.7) | 0.306 |
| IVUS | 452 (20.4) | 61 (25.1) | 0.086 | 617 (23.7) | 66 (21.6) | 0.401 | 766 (20.8) | 92 (22.1) | 0.549 |
| OCT | 16 (0.7) | 1 (0.4) | 0.580 | 27 (1.0) | 1 (0.3) | 0.354 | 24 (0.7) | 2 (0.5) | 0.675 |
| FFR | 25 (1.1) | 2 (0.8) | 0.665 | 36 (1.4) | 2 (0.7) | 0.425 | 54 (1.5) | 2 (0.5) | 0.119 |
| Drug-eluting stents |  |  |  |  |  |  |  |  |  |
| ZES, *n* (%) | 696 (31.4) | 82 (33.7) | 0.454 | 861 (33.1) | 124 (40.5) | 0.009 | 1217 (33.1) | 164 (39.3) | 0.010 |
| EES, *n* (%) | 1108 (50.0) | 119 (49.0) | 0.766 | 1322 (50.8) | 137 (44.8) | 0.046 | 1871 (50.8) | 203 (48.7) | 0.409 |
| BES, *n* (%) | 368 (16.6) | 39 (16.0) | 0.827 | 360 (13.8) | 38 (12.4) | 0.539 | 500 (13.6) | 43 (10.3) | 0.062 |
| Others, *n* (%) | 45 (2.0) | 3 (1.2) | 0.395 | 58 (2.2) | 7 (2.3) | 0.949 | 94 (2.6) | 7 (1.7) | 0.321 |
| Stent diameter (mm) | 3.16 ± 0.42 | 3.15 ± 0.47 | 0.603 | 3.16 ± 0.42 | 3.09 ± 0.40 | 0.005 | 3.11 ± 0.42 | 3.05 ± 0.41 | 0.006 |
| Stent length (mm) | 27.5 ± 11.7 | 26.3 ± 10.9 | 0.111 | 26.8 ± 11.2 | 26.0 ± 10.9 | 0.257 | 27.5 ± 11.9 | 26.9 ± 11.3 | 0.339 |
| Number of stent | 1.42 ± 0.74 | 1.44 ± 0.74 | 0.605 | 1.46 ± 0.78 | 1.46 ± 0.74 | 0.885 | 1.55 ± 0.82 | 1.59 ± 0.84 | 0.268 |

Values are means ± SD or numbers and percentages or median (quartiles 1–3). The *p* values for continuous data obtained from the analysis of variance. The *p* values for categorical data from chi-square or Fisher’s exact test. LVEF, left ventricular ejection fraction; BMI, body mass index; SBP, systolic blood pressure; DBP, diastolic blood pressure; STEMI, ST-elevation myocardial infarction; NSTEMI, non-STEMI; PCI, percutaneous coronary intervention; CABG, coronary artery bypass graft; CVA, cerebrovascular accident; HF, heart failure; CK-MB, creatine kinase myocardial band; NT-ProBNP, N-terminal probrain natriuretic peptide; hs-CRP, high-sensitivity C-reactive protein; eGFR, estimated glomerular filtration rate; HDL, high-density lipoprotein; LDL, low-density lipoprotein; CCBs, calcium channel blockers; IRA, infarct-related artery; LAD, left anterior descending coronary artery; LCx, left circumflex coronary artery; RCA, right coronary artery; CAD, coronary artery disease; ACC/AHA, American College of Cardiology/American Heart Association; IVUS, intravascular ultrasound; OCT, optical coherence tomography; FFR, fractional flow reserve; ZES, zotarolimus-eluting stent; EES, everolimus-eluting stent; BES, biolimus-eluting stent.

**Table S2.** Baseline characteristics of BB+RASI nonusers.

| **Variables** | **Normoglycemia (Group A2, *n* = 243)** | **Prediabetes**  **(Group B2, *n* = 306)** | Diabetes  (Group C2, *n* = 417) | p value | | | |
| --- | --- | --- | --- | --- | --- | --- | --- |
|  |  |  |  | Group A2  vs. Group B2 | Group A2  vs. Group C2 | Group B2  vs. Group C2 | Group A2  vs. Group B2  vs. Group C2 |
| **Male, *n* (%)** | **190 (78.2)** | **209 (68.3)** | 267 (64.0) | 0.010 | <0.001 | 0.231 | 0.001 |
| Age (years) | 64.3 ± 13.4 | 66.7 ± 13.4 | 67.2 ± 11.8 | 0.042 | 0.005 | 0.566 | 0.016 |
| LVEF (%) | 49.9 ± 13.1 | 49.7 ± 13.6 | 47.3 ± 13.7 | 0.826 | 0.015 | 0.020 | 0.017 |
| ≤40% | 43 (17.7) | 61 (19.9) | 117 (28.1) | 0.506 | 0.003 | 0.012 | 0.003 |
| BMI (kg/m^2^) | 23.4 ± 3.1 | 23.2 ± 3.4 | 23.9 ± 3.6 | 0.475 | 0.066 | 0.008 | 0.020 |
| SBP (mmHg) | 119.8 ± 25.1 | 122.8 ± 28.1 | 123.8 ± 29.5 | 0.194 | 0.070 | 0.659 | 0.216 |
| DBP (mmHg) | 74.7 ± 14.3 | 75.4 ± 15.8 | 75.4 ± 16.8 | 0.612 | 0.569 | 0.969 | 0.845 |
| STEMI, *n* (%) | 150 (61.7) | 185 (60.5) | 232 (55.6) | 0.762 | 0.126 | 0.195 | 0.232 |
| Primary PCI, *n* (%) | 143 (95.3) | 174 (94.1) | 218 (94.0) | 0.606 | 0.567 | 0.970 | 0.832 |
| NSTEMI, *n* (%) | 93 (38.3) | 121 (39.5) | 185 (44.4) | 0.762 | 0.126 | 0.195 | 0.232 |
| PCI within 24 hours | 78 (84.3) | 101 (83.2) | 153 (82.7) | 0.938 | 0.806 | 0.861 | 0.966 |
| Hypertension, *n* (%) | 83 (34.2) | 130 (42.5) | 259 (62.1) | 0.052 | <0.001 | <0.001 | <0.001 |
| Dyslipidemia, *n* (%) | 12 (4.9) | 32 (10.5) | 52 (12.5) | 0.018 | 0.002 | 0.414 | 0.007 |
| Previous MI, *n* (%) | 8 (3.3) | 12 (3.9) | 13 (3.1) | 0.820 | 0.902 | 0.681 | 0.835 |
| Previous PCI, *n* (%) | 10 (4.1) | 16 (5.2) | 27 (6.5) | 0.542 | 0.224 | 0.538 | 0.426 |
| Previous CABG, *n* (%) | 1 (0.4) | 0 (0.0) | 4 (1.0) | 0.443 | 0.657 | 0.142 | 0.199 |
| Previous CVA, *n* (%) | 17 (7.0) | 16 (5.2) | 45 (10.8) | 0.470 | 0.128 | 0.010 | 0.020 |
| Previous HF, *n* (%) | 1 (0.4) | 8 (2.6) | 12 (2.9) | 0.049 | 0.038 | 0.831 | 0.091 |
| Current smokers, *n* (%) | 118 (48.6) | 124 (40.5) | 132 (31.7) | 0.060 | <0.001 | 0.014 | <0.001 |
| Peak CK-MB (mg/dL) | 187.3 ± 278.8 | 163.6 ± 198.4 | 128.6 ± 158.8 | 0.263 | 0.003 | 0.011 | 0.001 |
| Peak troponin-I (ng/mL) | 59.8 ± 119.8 | 50.6 ± 89.1 | 61.2 ± 196.8 | 0.368 | 0.923 | 0.378 | 0.675 |
| Blood glucose (mg/dL) | 150.4 ± 67.4 | 157.2 ± 58.0 | 245.3 ± 119.4 | <0.001 | <0.001 | <0.001 | <0.001 |
| Hemoglobin A1c (%) | 5.30 ± 0.62 | 5.98 ± 0.21 | 7.88 ± 3.40 | <0.001 | <0.001 | <0.001 | <0.001 |
| NT-ProBNP (pg/mL) | 299.0 (58.0-2645.0) | 467.0 (101.5-2229.0) | 1124.0 (163.5-6746.5) | 0.389 | 0.001 | <0.001 | <0.001 |
| hs-CRP (mg/dL) | 13.2 ± 56.5 | 15.4 ± 49.3 | 22.4 ± 66.0 | 0.674 | 0.103 | 0.158 | 0.183 |
| Serum creatinine (mg/L) | 1.04 ± 0.44 | 1.35 ± 3.95 | 1.45 ± 1.58 | 0.178 | <0.001 | 0.649 | 0.109 |
| eGFR (mL/min/1.73 m^2^) | 84.1 ± 31.8 | 84.4 ± 37.8 | 74.8 ± 37.5 | 0.924 | 0.001 | 0.001 | <0.001 |
| <60 mL/min/1.73 m^2^ | 53 (21.8) | 66 (21.6) | 148 (35.5) | 0.954 | <0.001 | 0.001 | <0.001 |
| Total cholesterol (mg/dL) | 172.1 ± 42.5 | 184.6 ± 46.2 | 172.5 ± 48.4 | 0.001 | 0.911 | 0.001 | 0.001 |
| Triglyceride (mg/L) | 102.8 ± 67.4 | 111.1 ± 85.1 | 141.0 ± 117.7 | 0.154 | <0.001 | <0.001 | <0.001 |
| HDL cholesterol (mg/L) | 45.5 ± 25.7 | 44.2 ± 20.3 | 41.0 ± 14.8 | 0.517 | 0.013 | 0.020 | 0.010 |
| LDL cholesterol (mg/L) | 109.9 ± 41.6 | 120.2 ± 39.9 | 104.6 ± 41.7 | 0.004 | 0.122 | <0.001 | <0.001 |
| Diabetes management |  |  |  |  |  |  |  |
| Diet |  |  | 28 (6.7) |  |  |  |  |
| Oral agent |  |  | 266 (63.8) |  |  |  |  |
| Insulin |  |  | 27 (6.5) |  |  |  |  |
| untreated |  |  | 96 (23.0) |  |  |  |  |
| Discharge medications |  |  |  |  |  |  |  |
| Aspirin, *n* (%) | 218 (89.7) | 266 (86.9) | 367 (88.0) | 0.316 | 0.506 | 0.663 | 0.605 |
| Clopidogrel, *n* (%) | 200 (82.3) | 264 (86.3) | 344 (82.5) | 0.202 | 0.951 | 0.170 | 0.322 |
| Ticagrelor, *n* (%) | 20 (8.2) | 22 (7.2) | 34 (8.2) | 0.649 | 0.972 | 0.675 | 0.867 |
| Prasugrel, *n* (%) | 8 (3.3) | 12 (3.9) | 27 (6.5) | 0.820 | 0.104 | 0.182 | 0.121 |
| Cilostazole, *n* (%) | 23 (9.5) | 38 (12.4) | 44 (10.6) | 0.339 | 0.656 | 0.434 | 0.523 |
| CCBs, *n* (%) | 21 (8.6) | 25 (8.2) | 29 (7.0) | 0.878 | 0.429 | 0.539 | 0.700 |
| Lipid lowering agents | 190 (78.2) | 247 (80.7) | 340 (81.5) | 0.465 | 0.297 | 0.782 | 0.573 |
| IRA |  |  |  |  |  |  |  |
| Left main, *n* (%) | 7 (2.9) | 8 (2.6) | 15 (3.6) | 0.849 | 0.623 | 0.525 | 0.733 |
| LAD, *n* (%) | 118 (48.6) | 150 (49.0) | 207 (49.6) | 0.915 | 0.809 | 0.869 | 0.963 |
| LCx, *n* (%) | 39 (16.0) | 42 (13.7) | 62 (14.9) | 0.469 | 0.684 | 0.748 | 0.747 |
| RCA, *n* (%) | 79 (32.5) | 106 (34.6) | 133 (31.9) | 0.600 | 0.870 | 0.437 | 0.731 |
| Treated vessel |  |  |  |  |  |  |  |
| Left main, *n* (%) | 11 (4.5) | 13 (4.2) | 21 (5.0) | 0.874 | 0.852 | 0.723 | 0.878 |
| LAD, *n* (%) | 138 (56.8) | 168 (54.9) | 246 (59.0) | 0.658 | 0.580 | 0.272 | 0.543 |
| LCx, *n* (%) | 51 (21.0) | 77 (25.2) | 114 (27.3) | 0.265 | 0.077 | 0.512 | 0.192 |
| RCA, *n* (%) | 91 (37.4) | 118 (38.6) | 161 (38.6) | 0.860 | 0.803 | 0.990 | 0.951 |
| ACC/AHA lesion type |  |  |  |  |  |  |  |
| Type B1, *n* (%) | 25 (10.3) | 32 (10.5) | 57 (13.7) | 0.948 | 0.223 | 0.209 | 0.292 |
| Type B2, *n* (%) | 59 (24.3) | 94 (30.7) | 103 (24.7) | 0.104 | 0.926 | 0.073 | 0.128 |
| Type C, *n* (%) | 118 (48.6) | 121 (69.5) | 191 (45.8) | 0.034 | 0.494 | 0.093 | 0.084 |
| Extent of CAD |  |  |  |  |  |  |  |
| 1-vessel, *n* (%) | 139 (57.2) | 151 (49.3) | 170 (40.8) | 0.073 | <0.001 | 0.024 | <0.001 |
| 2-vessel, *n* (%) | 69 (28.4) | 93 (30.4) | 140 (33.6) | 0.610 | 0.193 | 0.356 | 0.355 |
| ≥3-vessel, *n* (%) | 35 (14.4) | 62 (20.3) | 107 (25.7) | 0.074 | 0.001 | 0.090 | 0.003 |
| IVUS | 61 (25.1) | 66 (21.6) | 92 (22.1) | 0.360 | 0.372 | 0.927 | 0.571 |
| OCT | 1 (0.4) | 1 (0.3) | 2 (0.5) | 0.870 | 0.900 | 0.752 | 0.951 |
| FFR | 2 (0.8) | 2 (0.7) | 2 (0.5) | 0.817 | 0.628 | 0.755 | 0.860 |
| Drug-eluting stents |  |  |  |  |  |  |  |
| ZES, *n* (%) | 82 (33.7) | 124 (40.5) | 164 (39.3) | 0.103 | 0.157 | 0.746 | 0.228 |
| EES, *n* (%) | 119 (49.0) | 137 (44.8) | 203 (48.7) | 0.327 | 0.943 | 0.327 | 0.507 |
| BES, *n* (%) | 39 (16.0) | 38 (12.4) | 43 (10.3) | 0.265 | 0.031 | 0.375 | 0.098 |
| Others, *n* (%) | 3 (1.2) | 7 (2.3) | 7 (1.7) | 0.524 | 0.753 | 0.593 | 0.639 |
| Stent diameter (mm) | 3.15 ± 0.47 | 3.09 ± 0.40 | 3.05 ± 0.41 | 0.136 | 0.007 | 0.170 | 0.016 |
| Stent length (mm) | 26.3 ± 10.9 | 26.0 ± 10.9 | 26.9 ± 11.3 | 0.753 | 0.524 | 0.301 | 0.567 |
| Number of stent | 1.44 ± 0.74 | 1.46 ± 0.74 | 1.59 ± 0.84 | 0.814 | 0.018 | 0.023 | 0.022 |

Values are means ± SD or numbers and percentages or median (quartiles 1–3). The *p* values for continuous data obtained from the analysis of variance. The *p* values for categorical data from chi-square or Fisher’s exact test. NSTEMI, non-STEMI; PCI, percutaneous coronary intervention; CABG, coronary artery bypass graft; CVA, cerebrovascular accident; HF, heart failure; CK-MB, creatine kinase myocardial band; NT-ProBNP, N-terminal probrain natriuretic peptide; hs-CRP, high-sensitivity C-reactive protein; eGFR, estimated glomerular filtration rate; HDL, high-density lipoprotein; LDL, low-density lipoprotein; CCBs, calcium channel blockers; IRA, infarct-related artery; LAD, left anterior descending coronary artery; LCx, left circumflex coronary artery; RCA, right coronary artery; CAD, coronary artery disease; ACC/AHA, American College of Cardiology/American Heart Association; IVUS, intravascular ultrasound; OCT, optical coherence tomography; FFR, fractional flow reserve; ZES, zotarolimus-eluting stent; EES, everolimus-eluting stent; BES, biolimus-eluting stent.

**Table S3.** Independent predictors for MACEs and HHF in BB+RASI users at two-year follow-up.

|  | **MACEs** | | | | **HHF** | | | |
| --- | --- | --- | --- | --- | --- | --- | --- | --- |
|  | **Unadjusted** | | **Adjusted** | | **Unadjusted** | | **Adjusted** | |
| **Variables** | **HR (95% CI)** | ***p* value** | **HR (95% CI)** | ***p* value** | **HR (95% CI)** | ***p* value** | **HR (95% CI)** | ***p* value** |
| Group A1 vs. Group B1 | 1.252 (0.983–1.594) | 0.069 | 1.299 (1.021–1.684) | 0.030 | 1.129 (0.501–2.348) | 0.769 | 1.252 (0.697–2.402) | 0.442 |
| Group A1 vs. Group C1 | 1.636 (1.315–2.036) | <0.001 | 1.363 (1.086–1.704) | 0.007 | 1.625 (0.806–3.275) | 0.175 | 1.431 (0.824–2.484) | 0.203 |
| Group B1 vs. Group C1 | 1.313 (1.087–1.587) | 0.005 | 1.126 (0.927–1.368) | 0.232 | 1.428 (0.760–2.685) | 0.268 | 1.131 (0.789–1.563) | 0.547 |
| Age, ≥65 years | 1.384 (1.176–1.629) | <0.001 | 1.078 (0.897–1.293) | 0.431 | 3.503 (2.398–5.117) | <0.001 | 2.310 (1.527–3.402) | <0.001 |
| Male sex | 1.449 (1.217–1.726) | <0.001 | 1.305 (1.077–1.582) | 0.007 | 2.018 (1.441–2.827) | <0.001 | 1.430 (1.001–2.084) | 0.050 |
| STEMI | 1.315 (1.118–1.548) | 0.001 | 1.234 (1.043–1.459) | 0.014 | 1.566 (1.124–2.183) | 0.008 | 1.501 (1.062–2.123) | 0.021 |
| LVEF, <40% | 1.833 (1.481–2.267) | <0.001 | 1.634 (1.302–2.018) | <0.001 | 8.581 (6.158–11.96) | <0.001 | 6.923 (4.897–9.832) | <0.001 |
| Hypertension | 1.244 (1.056–1.466) | 0.009 | 1.047 (0.880–1.244) | 0.607 | 1.833 (1.295–2.594) | 0.001 | 1.306 (0.971–1.757) | 0.077 |
| Clopidogrel | 1.244 (0.956–1.621) | 0.105 | 1.172 (0.895–1.534) | 0.249 | 1.226 (0.801–1.878) | 0.348 | 1.514 (0.876–2.365) | 0.054 |
| Lipid-lowering agents | 1.547 (1.205–1.986) | 0.001 | 1.400 (1.087–1.803) | 0.009 | 1.302 (0.684–2.477) | 0.421 | 1.901 (1.024–3.512) | 0.066 |
| eGFR, <60 mL/min/1.73 m^2^ | 1.661 (1.372–2.009) | <0.001 | 1.412 (1.111–1.810) | 0.001 | 4.338 (3.113–6.045) | <0.001 | 2.801 (1.995–3.974) | <0.001 |
| ACC/AHA type B2/C lesion | 1.103 (0.903–1.347) | 0.337 | 1.116 (0.872–1.402) | 0.385 | 1.838 (1.113–2.982) | 0.014 | 1.875 (1.123–2.938) | 0.024 |
| ≥3-vessel disease | 1.908 (1.601–2.273) | <0.001 | 1.704 (1.424–2.039) | <0.001 | 1.449 (0.992–2.116) | 0.055 | 1.231 (0.885–1.932) | 0.317 |
| Stent length >30 mm | 1.135 (0.953–1.353) | 0.155 | 1.982 (1.114–3.782) | 0.034 | 2.081 (0.515–8.404) | 0.304 | 2.031 (1.533–2.691) | 0.302 |

BB, ß-blockers; RASI, renin–angiotensin system inhibitors; HR, hazard ratio; CI, confidence interval; MACEs, major adverse cardiac events; HHF, hospitalization for heart failure; STEMI, ST-elevation myocardial infarction; LVEF, left ventricular ejection fraction; eGFR, estimated glomerular filtration rate; ACC/AHA, American College of Cardiology/American Heart Association; IVUS, intravascular ultrasound.
